# Ultrasound-Guided Minimally Invasive Interventions for Temporomandibular Joint Disorders: A Scoping Review

**DOI:** 10.3390/jcm15145454

**Published:** 2026-07-12

**Authors:** Tomasz Horodniczy, Klaudia Kwiatkowska, Kamila Chęcińska, Maciej Chęciński, Karolina Grzybowska-Kowalczyk, Wojciech Macek, Maja Kosińska, Amelia Hoppe, Julia Kasprzycka, Oliwia Jagiełło, Zuzanna Baniak, Maciej Sikora

**Affiliations:** 1 Ortho.pl Dental Office, Buforowa 34, 52-131 Wrocław, Poland; tomasz.horodniczy2@gmail.com; 2 National Medical Institute of the Ministry of the Interior and Administration, Wołoska 137, 02-507 Warsaw, Poland; lek.dent.k.kwiatkowska@wp.pl (K.K.); maciej.checinski@pimmswia.gov.pl (M.C.); maciej.sikora@pimmswia.gov.pl (M.S.); 3 Department of Maxillofacial Surgery, Hospital of the Ministry of the Interior and Administration, Wojska Polskiego 51, 25-375 Kielce, Poland; 4 Uśmiech Family Dental Clinic, Nastrojowa 26, 91-496 Łódź, Poland; karolina.orto.gk@gmail.com; 5 Department of Oral Surgery, Preventive Medicine Center, Komorowskiego 12, 30-106 Kraków, Poland; ld.wojciech.macek@wp.pl (W.M.); majaakosinska@gmail.com (M.K.); amelia.a.hoppe@gmail.com (A.H.); 6 Miodowa Clinic, Głowaccy Medical and Dental Practice Professional Partnership, Miodowa 2, 62-090 Kiekrz, Poland; 7 Gabinety Lekarskie Dent-im M.B. Pawelczyk Spółka Jawna, Różana 13/1, 61-577 Poznań, Poland; oliwiajagiellodent@gmail.com; 8 Stomatologia Centrum, Moniuszki 3/7, 32-020 Wieliczka, Poland; baniakzuzanna@gmail.com; 9 Department of Biochemistry and Medical Chemistry, Pomeranian Medical University, Powstańców Wielkopolskich 72, 70-111 Szczecin, Poland

**Keywords:** ultrasound-guided interventions, ultrasonography, intra-articular injection, arthrocentesis, platelet-rich plasma, minimally invasive treatment, image-guided procedures, needle placement accuracy, scoping review

## Abstract

**Background/Objectives:** Ultrasound is increasingly used to guide minimally invasive interventions for temporomandibular joint (TMJ) disorders because it enables real-time visualization of the joint, surrounding tissues, and needle position. This scoping review aimed to map and characterize the available evidence on ultrasound-guided minimally invasive interventions for temporomandibular disorders (TMDs). **Methods:** The review followed PRISMA-ScR guidelines and was registered in the Open Science Framework (osf.io/r42m5). PubMed/MEDLINE, BASE, and Europe PMC were searched up to 4 April 2026. Original clinical and technical studies evaluating ultrasound-guided minimally invasive interventions for TMDs were included. **Results:** Seventeen studies published between 2012 and 2026 met the eligibility criteria. Ultrasound guidance was used mainly for intra-articular injections, arthrocentesis, and botulinum toxin injections into the lateral pterygoid muscle. Most studies reported reductions in pain and improvements in jaw function. Compared with conventional approaches, several included studies reported more accurate needle placement, fewer insertion attempts, less need for needle repositioning, and shorter procedure times when ultrasound guidance was used. However, these procedural advantages did not consistently translate into superior long-term clinical outcomes. **Conclusions:** Overall, ultrasound-guided minimally invasive TMJ interventions appear feasible and generally safe, with their main benefit being improved visualization and procedural precision. Further standardized, high-quality studies are needed to clarify their clinical impact.

## 1. Introduction

### 1.1. Rationale

The increasing integration of imaging modalities into clinical practice has reshaped the way minimally invasive interventions are performed in musculoskeletal medicine [1]. In the field of temporomandibular disorders (TMDs), where anatomical complexity and limited access to the joint space present procedural challenges, ultrasound guidance has emerged as a promising tool to enhance both diagnostic accuracy and therapeutic precision [2,3].

Traditionally, intra-articular and periarticular interventions within the temporomandibular joint (TMJ) have been performed using landmark-based techniques, which rely heavily on clinician experience and may result in variability in needle placement [4]. The introduction of ultrasound guidance offers real-time visualization of soft tissues, joint structures, and needle trajectory, potentially improving targeting accuracy and reducing the risk of complications [4,5]. In addition, ultrasound enables dynamic assessment of joint function, which may further support individualized treatment approaches [2,6]. Moreover, ultrasound is a rapid, non-invasive, relatively low-cost, repeatable, and widely available technique, making it particularly suitable for outpatient settings and for repeated, real-time monitoring of interventional procedures.

Despite these advantages, ultrasound also has important limitations. Deeper or more posteriorly located structures may be challenging to visualize, acoustic shadowing from bony contours can obscure relevant anatomy, and both image acquisition and interpretation are highly operator-dependent. Differences in examiner experience, equipment quality, and scanning protocols may therefore lead to variability in image quality, reporting, and clinical decision-making. In the context of TMJ interventions, these constraints highlight the need for standardized scanning approaches, clear reporting of ultrasound findings, and dedicated training to ensure that image-guided procedures are performed safely and reproducibly.

A growing number of studies have explored the application of ultrasound guidance in various minimally invasive procedures for TMDs, including intra-articular injections, lavage techniques, and other interventional strategies [7]. However, this body of literature remains heterogeneous, encompassing a wide range of procedural techniques, operator-dependent factors, and clinical indications [8]. Furthermore, terminology and reporting standards are not uniform, which complicates efforts to synthesize and interpret the available evidence [7,9]. Evidence from compartment-specific TMJ injection research suggests that the exact intra-articular location of injectate deposition has a meaningful impact on clinical outcomes. In a recent systematic review and meta-analysis [10], inferior-compartment injections were shown to provide greater improvements in articular pain, Helkimo index, and maximum mouth opening compared with injections limited to the superior compartment. These findings underscore the clinical relevance of accurate compartment-specific targeting within the TMJ and suggest that imaging-guided techniques may facilitate more precise needle placement. Furthermore, the subsequent rapid review of temporomandibular joint injection techniques [11] synthesized evidence from contemporary randomized controlled trials evaluating intra-articular therapies. These included arthrocentesis-based protocols as well as injections of platelet-derived preparations, hyaluronic acid, non-steroidal anti-inflammatory drugs, and hypertonic dextrose. The review outlined current evidence and highlighted emerging directions in minimally invasive TMJ injection therapy.

### 1.2. Objectives

The objective of this scoping review was to map the extent, range, and characteristics of the available evidence on ultrasound-guided minimally invasive interventions for TMDs, to describe the types of procedures and outcomes reported, and to identify gaps in the literature.

## 2. Materials and Methods

### 2.1. Protocol and Registration

The scoping review was performed and reported following the PRISMA-ScR guidelines and checklist [12]. The review was not designed to answer a narrowly focused clinical effectiveness question or to perform a meta-analysis, but rather to provide a descriptive overview of the available evidence in accordance with the aims of a scoping review. A review protocol was developed prior to study initiation and registered in the Open Science Framework (OSF) repository under registration number: osf.io/r42m5.

### 2.2. Eligibility Criteria

Eligibility criteria for this scoping review are summarized in Table 1. Briefly, eligible records included original clinical studies, case series, case reports, and technical or feasibility reports describing ultrasound-guided minimally invasive interventions for temporomandibular disorders (TMDs), including procedures directed at the temporomandibular joint (TMJ), periarticular tissues, or functionally related masticatory muscles. Studies limited to diagnostic ultrasound, non-ultrasound-guided procedures, non-human research, reviews, editorials, and conference abstracts without full data were excluded. No language restrictions were applied, and studies published up to 4 April 2026 were considered.

### 2.3. Information Sources

Information for this scoping review was drawn from a focused set of bibliographic and academic search sources selected to cover both biomedical literature and broader open-access academic resources relevant to ultrasound-guided interventions for temporomandibular disorders. Electronic searches were conducted in MEDLINE via PubMed, BASE, and Europe PMC. PubMed is a free resource developed and maintained by the National Center for Biotechnology Information at the U.S. National Library of Medicine, National Institutes of Health, and contains more than 40 million citations and abstracts of biomedical and life sciences literature [13]. BASE is a multidisciplinary academic search engine operated by Bielefeld University Library, indexing more than 400 million records from more than 12,000 content providers, including journals, institutional repositories, digital collections, theses, reports, conference materials, and other open-access academic resources [14]. Its inclusion was intended to broaden the search beyond conventional biomedical databases and to capture relevant grey literature. Europe PMC provides free access to life sciences literature from trusted sources, including journal articles, preprints, books, reviews, protocols, and other documents enriched with links to supporting data and related resources [15].

### 2.4. Search

A comprehensive search strategy was developed and piloted in PubMed. It combined controlled vocabulary and free-text terms to capture concepts relating to the temporomandibular joint and ultrasound-guided minimally invasive interventions (synonyms and spelling variants for the joint, ultrasound guidance, and procedural terms). Final searches were run in PubMed (MEDLINE), BASE, and Europe PMC and were adapted to each platform’s syntax and indexing. The core search query used across databases was: “(“temporomandibular joint” OR TMJ OR temporomandibular) AND (ultrasound OR ultrasonography OR sonography OR “ultrasound-guided” OR “sonography-guided”) AND (injection OR injections OR arthrocentesis OR aspiration OR lavage OR “joint lavage” OR arthroscopy OR “minimally invasive” OR aspirat* OR “platelet-rich plasma” OR PRP OR hyaluronic OR steroid* OR “local anesthetic”) NOT (review OR “systematic review” OR “meta-analysis”)”.

Final searches were run in PubMed/MEDLINE, BASE, and Europe PMC, adapted to each platform’s syntax and indexing, and completed on 4 April 2026.

### 2.5. Selection of Sources of Evidence

All records identified through the database search were imported into Rayyan screening software, it was accessed on 4 April 2026 [16], and duplicates were removed (T.H.). Study selection was conducted in two stages. Before formal screening, the reviewers completed a calibration exercise to ensure consistent application of the eligibility criteria. First, titles and abstracts were independently reviewed in a blinded manner to identify potentially relevant studies (J.K. and O.J.). Subsequently, the full texts of the selected articles were independently assessed for eligibility against the predefined inclusion and exclusion criteria (J.K. and O.J.). Inter-rater agreement was assessed using Cohen’s kappa coefficient. Agreement was substantial at both the title and abstract screening stage (κ = 0.90) and the full-text review stage (κ = 0.94). Any disagreements were resolved through discussion and consensus. Reasons for exclusion at the full-text stage were recorded. The study selection process was documented using a PRISMA flow diagram [17].

### 2.6. Data Charting Process

Data from the included studies were charted using a standardized data charting form developed by the research team. The form was pilot tested on a small sample of studies and refined as necessary. Two reviewers independently charted data from the selected articles using tables prepared in Google Sheets to ensure accuracy and consistency (J.K. and O.J.). Any discrepancies were resolved through discussion and consensus between the reviewers.

### 2.7. Data Items

The following data items were charted from each included study using the standardized data charting form: study design, sample size, participant characteristics, type of temporomandibular joint disorder, intervention type, injected agent (when applicable), ultrasound technical parameters (transducer type and frequency, patient positioning, and needle approach), operator characteristics, comparator, and follow-up duration.

Technical outcomes included needle or cannula placement accuracy, number of needle passes, and procedure duration. Clinical outcomes included pain intensity, mandibular function, and maximum mouth opening. Additional data extracted comprised adverse events and complications, patient-reported global improvement, duration of treatment effect, requirement for repeat interventions, and the main findings of each study. Additional information relevant to the objectives of the review was recorded when available.

### 2.8. Critical Appraisal of Individual Sources of Evidence

The Joanna Briggs Institute (JBI) critical appraisal checklists appropriate for each study design were used: the JBI Checklist for Randomized Controlled Trials, the JBI Checklist for Quasi-Experimental Studies, the JBI Checklist for Cohort Studies, the JBI Checklist for Case Series, and the JBI Checklist for Text and Opinion Papers (for technical notes and reports).

This appraisal was included as a supplementary descriptive element to contextualize the evidence and did not change the methodological design of the study as a scoping review. The appraisal results were not used to exclude studies, but to contextualize the interpretation of findings. Evidence from studies with greater methodological limitations was interpreted more cautiously, and appraisal outcomes were considered when discussing the strength, consistency, and uncertainty of the available evidence.

### 2.9. Synthesis of Results

Because of the heterogeneity of the included studies in terms of intervention type, ultrasound protocols, study design, and reported outcomes, a quantitative synthesis was not considered appropriate. The results were therefore synthesized descriptively using a narrative mapping approach.

This approach is consistent with guidance provided in the Joanna Briggs Institute Manual for Evidence Synthesis, which recommends narrative and descriptive synthesis in scoping reviews when substantial methodological and clinical heterogeneity precludes meaningful quantitative pooling. In the present review, heterogeneity was observed across patient populations, intervention types, target anatomical structures, injected agents, study designs, and outcome measures [18].

The included studies were grouped according to the type of ultrasound-guided intervention performed, including intra-articular injections, arthrocentesis or lavage procedures, and interventions targeting the lateral pterygoid muscle. Extracted data were summarized in structured evidence tables and narratively synthesized to identify procedural patterns and areas of methodological heterogeneity across the available evidence base.

## 3. Results

### 3.1. Selection of Sources of Evidence

**Figure 1:**
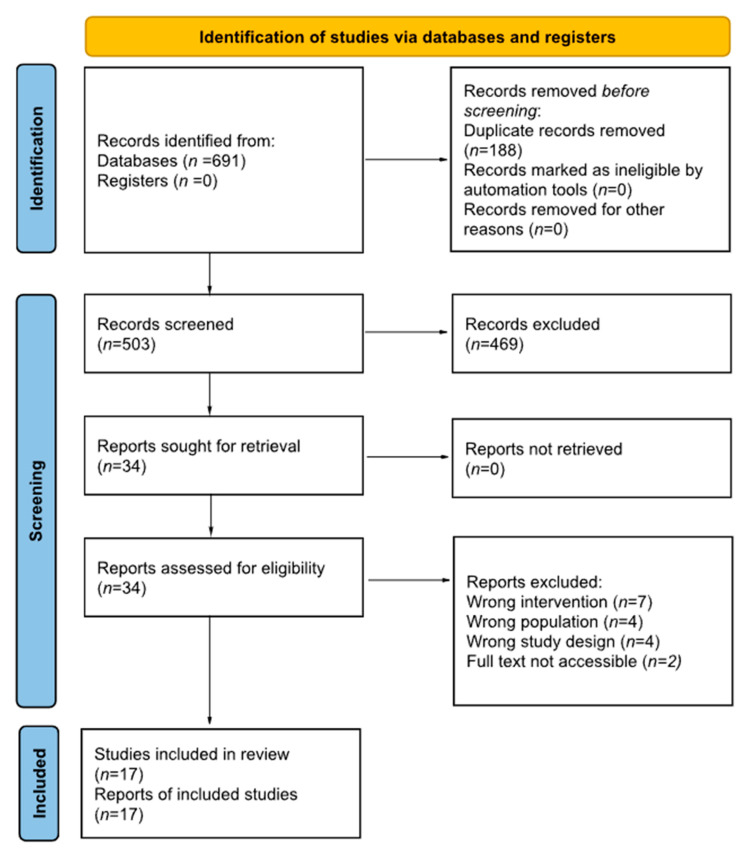
PRISMA flow diagram of study selection.

Reasons for exclusion at the full-text stage are presented in Table A1, and the overall study selection process is illustrated in the PRISMA flow diagram (Figure 1). Following screening and eligibility assessment, 17 studies met the inclusion criteria and were included in this scoping review.

### 3.2. Characteristics of Sources of Evidence

The 17 included sources of evidence were published between 2012 and 2026 and evaluated ultrasound-guided minimally invasive interventions for temporomandibular joint disorders. The included literature represented a heterogeneous body of evidence, including randomized clinical trials, prospective randomized or comparative studies, clinical follow-up studies, prospective cohort or clinical trial studies, case series, and technical notes or technical reports. Sample sizes varied substantially, ranging from small technical reports or case series involving two to four patients to larger comparative studies including up to 100 participants.

The eligible studies investigated a broad spectrum of temporomandibular joint disorders. The most frequently represented indications were internal derangement, disc displacement with or without reduction, acute closed lock, Wilkes stage III or higher temporomandibular disorders, and temporomandibular joint dysfunction refractory to conservative treatment. Other populations included patients with primary temporomandibular joint osteoarthritis, degenerative temporomandibular joint disease, chronic recurrent temporomandibular joint dislocation, oromandibular dystonia-related mandibular movement disorders, and children with juvenile idiopathic arthritis involving the temporomandibular joint.

The ultrasound-guided interventions were diverse. Injection-based procedures included intra-articular corticosteroid injection, hyaluronic acid injection, platelet-rich plasma injection, tenoxicam injection, dextrose prolotherapy, autologous blood injection, and botulinum toxin type A injection. Botulinum toxin was injected into the lateral pterygoid muscle in studies involving chronic or recurrent dislocation, oromandibular dystonia, or anterior disc displacement with reduction. Several studies evaluated ultrasound-guided arthrocentesis, including conventional two-needle arthrocentesis, single-puncture arthrocentesis, modified double-lumen or double-barrel techniques, and approaches designed to access either the upper joint space, the lower joint space, or both temporomandibular joint compartments. Detailed characteristics of the included studies are presented in Table 2.

Ultrasound-related procedural details were variably reported. Where specified, studies used linear probes, with reported frequencies ranging from 5–12 MHz to 18 MHz. Several studies described probe positioning in the preauricular region, commonly perpendicular to the zygomatic arch and parallel to the mandibular ramus. Some protocols used mouth opening and closing to identify the joint space, whereas others performed the procedure in a closed-mouth or open-mouth position, depending on the target joint compartment or intervention. Doppler imaging was reported in some studies to identify surrounding neurovascular structures. In several articles, however, transducer frequency, detailed ultrasound settings, or standardized patient positioning were not clearly described. Technical outcomes were reported inconsistently and ranged from qualitative descriptions of feasibility to quantitative measures such as first-attempt placement success, number of needle manipulations, and procedure duration. A summary of ultrasound-related procedural characteristics is provided in Table 3.

Comparators were included in several studies. These most commonly consisted of conventional landmark-based arthrocentesis or non-guided injection techniques. Some studies compared different ultrasound-guided injectates, such as hyaluronic acid, corticosteroid, tenoxicam, saline, platelet-rich plasma, or triamcinolone acetonide. Other studies were non-comparative and focused on feasibility, safety, or clinical outcomes following a single ultrasound-guided procedure.

### 3.3. Critical Appraisal Within Sources of Evidence

Methodological quality varied substantially across studies, with randomized and comparative studies generally achieving higher appraisal scores than case series and technical reports. Detailed appraisal results are presented in Table A2, Table A3, Table A4 and Table A5.

### 3.4. Results of Individual Sources of Evidence

The extracted outcomes varied between studies. Technical outcomes included needle placement, visualization of injectate distribution, the number of needle manipulation or insertion attempts, procedure duration, need for needle repositioning, and feasibility of accessing the intended joint compartment. Clinical outcomes most commonly included pain intensity, maximal mouth opening or interincisal distance, mandibular range of motion, chewing function, joint sounds, recurrence of dislocation, and patient-reported symptom improvement. Safety outcomes were inconsistently reported, ranging from explicit statements of no complications to descriptions of transient facial nerve paralysis, infection, hemorrhage, hematoma, trismus, injection-site scarring, or transient positive aspiration. Follow-up periods ranged from immediate postoperative or short-term assessments to 6 months, 1 year, or longer in selected studies. Overall, most studies reported improvements in pain and functional outcomes following ultrasound-guided interventions, although the magnitude of benefit and the outcome measures used varied substantially across studies. Reported adverse events were generally uncommon and predominantly transient, with no study reporting permanent procedure-related complications. A detailed summary of clinical outcomes, safety findings, and follow-up periods is presented in Table 4.

### 3.5. Synthesis of Results

Injection-based studies most commonly reported improvement in pain and/or mandibular function after ultrasound-guided treatment. Habibi et al. reported resolution of pre-injection pain in all symptomatic children after corticosteroid injection for juvenile idiopathic arthritis-related temporomandibular joint arthritis [19]. Gencer et al. found that ultrasound-guided hyaluronic acid injection resulted in greater pain relief than tenoxicam, betamethasone, or saline [20]. Al-Delayme et al. reported reduced pain and improved maximal mouth opening after platelet-rich plasma injection in patients with non-reducing disc displacement [23]. Similarly, Pandey et al. found that platelet-rich plasma produced more sustained pain and functional improvement than triamcinolone acetonide in primary temporomandibular joint osteoarthritis [26]. Kheder et al. reported pain improvement after dextrose prolotherapy in both ultrasound-guided and non-guided groups, with no significant difference between groups [21]. Overall, injection-based studies consistently reported reductions in pain and improvements in function, although comparative evidence remained limited.

Studies involving ultrasound-guided botulinum toxin injection into the lateral pterygoid muscle also reported favorable clinical outcomes. Lee et al. described improvement in recurrent dislocation or involuntary mandibular movements after intraoral botulinum toxin injection, with no significant complications [24]. Othman et al. reported significant improvements in pain, mouth opening, lateral excursion, clicking, and MRI disc position after botulinum toxin injection in patients with anterior disc displacement with reduction [28].

Arthrocentesis studies showed improvement in pain and/or mandibular function in both ultrasound-guided and conventional treatment groups. In comparative studies, reported differences most consistently concerned procedural parameters, whereas clear superiority in longer-term clinical outcomes was not consistently demonstrated. Bhargava et al. and Bahaa et al. reported fewer needle manipulations or insertion attempts and shorter procedure time with ultrasound guidance [22,32]. Antony et al. reported no need for needle repositioning in the ultrasound-guided group, compared with repositioning in 16 of 40 conventional cases, as well as lower pain on postoperative day 3 [33]. However, Sivri et al., Şentürk et al., Bhargava et al., Bahaa et al., and Antony et al. generally found no consistent significant between-group advantage in later pain or mouth-opening outcomes [22,29,32,33,34].

Non-comparative arthrocentesis studies and technical reports mainly supported the feasibility of ultrasound-guided temporomandibular joint access. Bilgir et al. reported significant short-term improvement in pain and mandibular movements after ultrasound-guided single-puncture arthrocentesis [31]. Dayisoylu et al. and Levorova et al. described feasible ultrasound-guided approaches for arthrocentesis or lower joint space injection, although structured clinical outcome data were limited or not reported [30,35]. Tesch et al. described a technique for ultrasound-guided access to both upper and lower joint compartments, established in cadavers and applied in two patients [27].

Safety reporting varied across the included studies. Several studies reported no complications, while others described isolated or transient events, including injection-site scarring, trismus, transient facial nerve paralysis, infection, hemorrhage or hematoma, intraoperative bleeding, and transient positive aspiration. Overall, the extracted studies most often reported clinical improvement after ultrasound-guided minimally invasive interventions, while comparative arthrocentesis studies more consistently demonstrated procedural benefits than sustained superiority in clinical outcomes.

## 4. Discussion

### 4.1. Summary of Evidence

The available evidence suggests that ultrasound guidance is increasingly used in minimally invasive interventions for temporomandibular joint disorders, particularly in arthrocentesis, intra-articular injections, and selected periarticular procedures. In the included studies, improvements in pain and mandibular function were frequently reported after ultrasound-guided interventions, although the strength of evidence varied substantially across study designs and clinical indications [2,7,31].

A consistent finding across comparative studies was that ultrasound guidance seemed to provide clearer procedural benefits than long-term clinical superiority. Bahaa et al. reported shorter procedure time or improved technical performance when ultrasound guidance was used, while Hu et al. similarly summarized that ultrasound-guided arthrocentesis may improve procedural aspects compared with conventional approaches [7,32,36]. However, differences in pain reduction and mouth-opening outcomes were less consistent. In addition, the relatively large superior joint compartment and diffusion of injected substances may reduce the clinical impact of small differences in needle position. Another contributing factor may be the limited statistical power of most available studies and the heterogeneity of patient populations and outcome measures. Therefore, improvements in procedural accuracy do not necessarily translate into measurable long-term clinical superiority [7,32,36].

This pattern is also consistent with broader musculoskeletal literature. Flores et al. emphasized that ultrasound-guided injection and aspiration of small joints can improve real-time visualization of the target area, needle trajectory, and surrounding structures, which is particularly relevant in anatomically narrow or difficult-to-access regions [4]. Mezian et al. similarly described ultrasound guidance as a useful tool in musculoskeletal interventions because it allows dynamic visualization and more controlled needle placement, while Hattori et al. highlighted the importance of image guidance in anatomically complex and interconnected joint spaces [37,38]. Hohenberger et al. showed that accurate puncture of the temporomandibular joint can be technically challenging, supporting the rationale for imaging assistance in procedures where precise intra-articular access is important [5]. Similar observations have been reported in other musculoskeletal interventions, where image guidance primarily improves procedural confidence and reproducibility rather than the intrinsic efficacy of the administered treatment. Consequently, ultrasound guidance should be viewed as a tool that enhances treatment delivery rather than as an intervention capable of independently modifying biological outcomes.

Injection-based studies showed generally favorable outcomes, but they were heterogeneous in terms of injected agents, diagnoses, and follow-up periods. Other studies explored hyaluronic acid, corticosteroids, dextrose prolotherapy, and biologic or orthobiologic approaches, reflecting the growing diversity of minimally invasive TMJ interventions [2,39]. This heterogeneity probably contributes to the inconsistent findings observed across studies. Because treatment effects may differ substantially depending on the injected agent and underlying pathology, it remains difficult to distinguish the specific contribution of ultrasound guidance from the therapeutic effect of the intervention itself.

Ultrasound guidance may be especially useful in procedures targeting anatomically demanding structures outside the joint space. Othman et al. reported improvement after ultrasound-guided botulinum toxin type A injection into the lateral pterygoid muscle in patients with anterior disc displacement with reduction [28]. Together, these findings suggest that ultrasound may be particularly valuable when the target is small, deep, or close to important anatomical structures.

Safety is another important consideration. However, the apparent low complication rate should be interpreted cautiously. Most studies were small, and adverse event reporting was inconsistent or incomplete. Consequently, the absence of major complications does not necessarily indicate superior safety. Larger studies with standardized safety reporting are required to determine whether improved anatomical visualization translates into a measurable reduction in procedure-related adverse events. Cömert Kiliç and Gözgeç highlighted that anatomical factors such as dehiscence of the roof of the glenoid fossa may influence the safety of intra-articular TMJ injections [40]. These observations suggest that ultrasound guidance may contribute to improved anatomical orientation during technically demanding procedures, although evidence supporting a reduction in complication rates remains limited.

Another important aspect is the operator-dependent nature of ultrasonography. Differences in examiner experience, familiarity with TMJ anatomy, procedural training, and ultrasound equipment may affect image acquisition, needle visualization, needle placement accuracy, and procedural reproducibility. Because most included studies provided limited information on operator training, learning curves, and procedural standardization, the generalizability of the reported outcomes to less experienced settings remains uncertain. Future studies should report operator experience and investigate the influence of training and equipment characteristics on procedural success and clinical outcomes.

Overall, the current evidence suggests that ultrasound-guided minimally invasive interventions for TMDs are feasible and may offer procedural advantages, especially in terms of visualization, targeting, and technical control. At the same time, the evidence does not yet consistently demonstrate clear superiority over conventional techniques in long-term clinical outcomes. Future studies should therefore use standardized ultrasound protocols, clearly defined diagnostic criteria, and comparable outcome measures to determine when ultrasound guidance provides meaningful clinical benefit rather than only technical improvement.

### 4.2. Strengths and Limitations

This review has several strengths, including a comprehensive search strategy, the inclusion of studies without language restrictions, and the systematic mapping of a heterogeneous body of evidence on ultrasound-guided minimally invasive interventions for TMDs. The inclusion of a critical appraisal of the primary studies provided additional context for interpreting the available evidence and identifying methodological gaps. The review also summarizes ultrasound-related technical aspects that are often inconsistently reported, including transducer characteristics, patient positioning, needle approaches, and Doppler use.

Several limitations should also be considered. The review was restricted to published studies, and some relevant unpublished or gray literature may not have been identified. The included studies were highly heterogeneous with respect to interventions, target structures, patient populations, ultrasound protocols, outcome measures, and follow-up periods, which limited comparability and precluded quantitative synthesis. In addition, the overall strength of the evidence was constrained by small sample sizes, variable methodological quality, and the predominance of observational studies, case series, and technical reports. Furthermore, the effectiveness and reproducibility of ultrasound-guided interventions are inherently operator dependent. Variations in training, procedural experience, familiarity with TMJ anatomy, and ultrasound equipment may influence image acquisition, needle placement accuracy, and clinical outcomes. The absence of standardized training pathways and competency reporting across studies limits assessment of external validity and may affect the translation of reported results into routine clinical practice.

The interpretation of the findings should be considered in light of the appraisal results, as several included studies had methodological limitations related to study design, sample size, comparator selection, blinding, or outcome reporting. Consequently, reported procedural or clinical benefits should be viewed as preliminary and hypothesis-generating rather than definitive evidence of effectiveness.

## 5. Conclusions

This scoping review identified a heterogeneous body of literature on ultrasound-guided minimally invasive interventions for TMDs. The included studies reported the use of ultrasound guidance for intra-articular injections, arthrocentesis or lavage procedures, and injections targeting the lateral pterygoid muscle. Reported findings suggest that ultrasound guidance may improve procedural visualization and needle placement in selected settings; however, the available evidence remains limited by heterogeneity in study design, intervention protocols, operator experience, comparators, and outcome reporting. Therefore, the current findings should be interpreted as preliminary and hypothesis-generating rather than definitive evidence of clinical superiority. Further well-designed, adequately powered, and standardized comparative studies are needed to clarify the clinical value, reproducibility, and safety of ultrasound-guided interventions for TMDs.

## Figures and Tables

**Table 1:** Eligibility criteria.

Category	Inclusion	Exclusion
Study type	Original clinical studies, case series, case reports, and technical/feasibility reports	Reviews, editorials, conference abstracts without full text, animal studies
Population	Patients with TMDs, including TMJ-related and muscle-related disorders	Non-human studies; studies unrelated to TMJ disorders
Intervention	Ultrasound-guided minimally invasive interventions for TMDs, including TMJ, periarticular, and masticatory muscle procedures	Diagnostic ultrasound only; non-ultrasound-guided procedures; studies using fluoroscopy, CT, MRI, or other imaging modalities for procedural guidance or needle confirmation
Comparator	Any comparator or none	Not applicable
Outcomes	Studies reporting technical, clinical, safety, or feasibility outcomes	No extractable outcome, safety, technical, or feasibility data
Language and date limits	No language restrictions; studies published up to 4 April 2026	Studies published after the search date

Abbreviations: TMDs, temporomandibular disorders; TMJ, temporomandibular joint; CT, computed tomography; MRI, magnetic resonance imaging. Ultrasound-guided procedures refer to interventions performed under real-time ultrasonographic guidance.

**Table 2:** Characteristics of included studies.

Author	Study Design	Sample Size	TMJ Disorder	Ultrasound-Guided Intervention	Comparator
Habibi et al., 2012 [19]	Retrospective clinical study	38 children (63 TMJs)	JIA-related TMJ arthritis	Corticosteroid injection	None
Gencer et al., 2014 [20]	RCT	100 patients	Degenerative TMJ disorders	HA, corticosteroid, tenoxicam, or saline injection	Different injectates
Kheder et al., 2024 [21]	RCT	22 patients (43 TMJs)	Disc displacement with reduction	Dextrose prolotherapy	Landmark-guided prolotherapy
Bhargava et al., 2019 [22]	RCT	20 patients	Acute closed lock/DDwoR	Single-puncture arthrocentesis	Conventional arthrocentesis
Al-Delayme et al., 2017 [23]	Prospective clinical trial	34 patients	Non-reducing disc displacement	PRP injection	None
Lee et al., 2024 [24]	Case series	4 patients	Recurrent dislocation/OMD	BTX injection into LPM	None
Gagnani et al., 2020 [25]	Prospective cohort study	19 patients	Chronic recurrent dislocation	Autologous blood injection	None
Pandey et al., 2025 [26]	RCT	38 patients	TMJ osteoarthritis	PRP injection	Triamcinolone injection
Tesch et al., 2024 [27]	Technical report	2 patients	DDwoR/DJD	Dual-compartment arthrocentesis/injection	None
Othman et al.,2026 [28]	Single-arm clinical trial	10 patients (20 TMJs)	ADDwR	BTX-A injection into LPM	None
Sivri et al., 2016 [29]	RCT	20 patients	Internal derangement	Arthrocentesis + HA	Conventional arthrocentesis
Levorova et al., 2015 [30]	Technical report	Not reported	Degenerative TMJ disease	Lower-compartment injection	None
Bilgir et al., 2020 [31]	Clinical follow-up study	40 patients (53 TMJs)	Wilkes stage III TMD	Single-puncture arthrocentesis	None
Bahaa et al., 2025 [32]	RCT	40 patients	DDwoR	SPA type 2 arthrocentesis + HA	Non-US-guided SPA
Antony et al., 2019 [33]	Comparative clinical study	80 patients	Internal derangement	Arthrocentesis	Conventional arthrocentesis
Şentürk et al., 2019 [34]	RCT	24 patients	Wilkes stage III TMD	SPA arthrocentesis	Non-guided SPA
Dayisoylu et al., 2013 [35]	Technical report	9 patients	TMJ disorders	Arthrocentesis	None

Abbreviations: ADDwR, anterior disc displacement with reduction; BTX, botulinum toxin; BTX-A, botulinum toxin type A; DDwoR, disc displacement without reduction; DJD, degenerative joint disease; HA, hyaluronic acid; JIA, juvenile idiopathic arthritis; LPM, lateral pterygoid muscle; OMD, oromandibular dystonia; PRP, platelet-rich plasma; RCT, randomized controlled trial; SPA, single-puncture arthrocentesis; TMD, temporomandibular disorder; TMJ, temporomandibular joint; US, ultrasound.

**Table 3:** Ultrasound-guided procedural characteristics.

Author	Ultrasound Equipment	Target Structure/Compartment	Procedural Approach	Technical Findings
Habibi et al., 2012 [19]	Not reported	TMJ space	Intra-articular injection	Visualization of needle placement and injectate distribution
Gencer et al., 2014 [20]	Not reported	Superior compartment	Intra-articular injection	No formal technical outcomes reported
Kheder et al., 2024 [21]	18 MHz linear probe + Doppler	TMJ capsule and masseter muscle	In-plane	Accurate single-puncture injection reported
Bhargava et al., 2019 [22]	12 MHz linear probe	Superior compartment	Single-puncture arthrocentesis	Fewer needle manipulations and shorter procedure time
Al-Delayme et al., 2017 [23]	Not reported	Superior compartment	Intra-articular injection	Technical success reported
Lee et al., 2024 [24]	E-CUBE 7	LPM	Intraoral approach	Real-time visualization of LPM and needle placement
Gagnani et al., 2020 [25]	Not reported	TMJ space	Intra-articular injection	Accuracy emphasized but not quantified
Pandey et al., 2025 [26]	5–12 MHz linear probe	TMJ space	Intra-articular injection	Successful intra-articular placement confirmed by test injection
Tesch et al., 2024 [27]	Butterfly iQ+ (1–10 MHz)	Superior and inferior compartments	Dual-compartment single-puncture approach	Reproducible access to both compartments
Othman et al., 2026 [28]	High-frequency linear probe + Doppler	LPM	In-plane	Successful guided delivery in all cases
Sivri et al., 2016 [29]	Linear probe	Superior compartment	Two-needle arthrocentesis	Longer procedure time than conventional technique
Levorova et al., 2015 [30]	7.5–14 MHz linear probe	Inferior compartment	Preauricular approach	Feasible lower-compartment access
Bilgir et al., 2020 [31]	6.5–10 MHz linear probe	Superior compartment	Y-cannula single-puncture arthrocentesis	Real-time monitoring of irrigation and cannula advancement
Bahaa et al., 2025 [32]	10–15 MHz linear probe	Superior compartment	Single-needle double-barrel cannula	Fewer insertion attempts and shorter procedure time
Antony et al., 2019 [33]	Linear probe	Superior compartment	Two-needle arthrocentesis	100% first-attempt placement; no needle repositioning
Şentürk et al., 2019 [34]	Linear probe	Superior compartment	Y-cannula SPA	Successful first-attempt cannula placement
Dayisoylu et al., 2013 [35]	7 MHz linear probe	Superior compartment	Two-needle arthrocentesis	Feasible and reproducible technique

Abbreviations: LPM, lateral pterygoid muscle; MHz, megahertz; SPA, single-puncture arthrocentesis; TMJ, temporomandibular joint.

**Table 4:** Clinical outcomes and safety.

Author	Pain Outcome	Functional Outcome (MMO/Function)	Safety	Follow-Up	Principal Study Conclusion
Habibi et al., 2012 [19]	Complete pain resolution in symptomatic patients	Improved chewing and jaw deviation	One injection-site scar	6–8 weeks	Safe and effective in JIA
Gencer et al., 2014 [20]	HA showed greatest pain reduction	Not reported	No major complications reported	6 weeks	HA superior to other injectates
Kheder et al., 2024 [21]	Significant pain reduction in both groups	Not reported	No complications	6 months	Similar clinical outcomes with and without US guidance
Bhargava et al., 2019 [22]	No between-group differences	Not reported	No complications	1 month	Technical advantages only
Al-Delayme et al., 2017 [23]	Significant pain reduction	Significant MMO improvement	No complications	6 months	PRP improved symptoms and function
Lee et al., 2024 [24]	Clinical improvement reported	Fewer dislocation episodes	No major complications	Up to 2 years	Effective BTX treatment
Gagnani et al., 2020 [25]	Not clearly reported	Not clearly reported	Not clearly reported	Not reported	Feasibility study
Pandey et al., 2025 [26]	PRP superior to steroid at 24 weeks	Greater MMO improvement with PRP	One transient trismus	24 weeks	PRP provided more durable outcomes
Tesch et al., 2024 [27]	Pain resolution reported	Functional improvement reported	One transient facial nerve palsy	6 months	Feasible dual-compartment technique
Othman et al., 2026 [28]	Significant pain reduction	Improved MMO and disc position	No complications	24 weeks	Effective BTX-A treatment
Sivri et al., 2016 [29]	Improvement in both groups	Improvement in both groups	No complications	3 months	No superiority over conventional arthrocentesis
Levorova et al., 2015 [30]	Not reported	Not reported	Not reported	Not reported	Technical feasibility study
Bilgir et al., 2020 [31]	Significant pain reduction	Improved mandibular mobility	Transient facial palsy, infection, hematoma	3 months	Clinically effective procedure
Bahaa et al., 2025 [32]	Improvement in both groups	Improvement in both groups	Minor intraoperative bleeding	12 weeks	Improved procedural efficiency
Antony et al., 2019 [33]	Lower early postoperative pain	Improved mouth opening	No major complications reported	1 month	Reduced needle repositioning
Şentürk et al., 2019 [34]	Improvement in both groups	Improvement in both groups	No complications	1 year	No clinical superiority over conventional SPA
Dayisoylu et al., 2013 [35]	Not reported	Not reported	Not reported	Not reported	Technical feasibility demonstrated

Abbreviations: BTX, botulinum toxin; BTX-A, botulinum toxin type A; HA, hyaluronic acid; JIA, juvenile idiopathic arthritis; MMO, maximum mouth opening; PRP, platelet-rich plasma; SPA, single-puncture arthrocentesis.

## Data Availability

Data are contained within the article.

## Abbreviations

The following abbreviations are used in this manuscript:

ADDwR	Anterior Disc Displacement with Reduction
BTX	Botulinum Toxin
BTX-A	Botulinum Toxin Type A
CT	Computed Tomography
DDwoR	Disc Displacement without Reduction
DJD	Degenerative Joint Disease
HA	Hyaluronic Acid
JBI	Joanna Briggs Institute
JIA	Juvenile Idiopathic Arthritis
LPM	Lateral Pterygoid Muscle
MHz	Megahertz
MMO	Maximum Mouth Opening
MRI	Magnetic Resonance Imaging
OMD	Oromandibular Dystonia
PRP	Platelet-Rich Plasma
RCT	Randomized Controlled Trial
SPA	Single-Puncture Arthrocentesis
TMD (s)	Temporomandibular Disorder (s)
TMJ	Temporomandibular Joint
US	Ultrasound
VAS	Visual Analogue Scale

## Appendix A

**Table A1:** Studies Excluded at Full-Text Assessment and Reasons for Exclusion.

Title		ID	Reason for Exclusion
Ultrasound-guided temporomandibular joint aspiration: technique and results in six cases of suspected septic arthritis	Symanski JS, 2023 [8]	PMID: 36348042	Wrong intervention (diagnostic arthrocentesis for suspected septic arthritis rather than therapeutic minimally invasive intervention for TMJ disorders)
Temporal mandibular joint chondrocalcinosis (tophaceous pseudogout) diagnosed by ultrasound-guided fine-needle aspiration	Fan J., 2019 [41]	PMID: 30908901	Wrong intervention (diagnostic fine-needle aspiration for TMJ pseudogout rather than therapeutic minimally invasive intervention for TMJ disorders)
Ultrasound-Guided Temporomandibular Joint Injection for Chronic Posthemimandibulectomy Jaw Pain	Chakraborty A., 2016 [42]	PMID: 27607407	Wrong population (no clear TMJ disorder diagnosis)
US-guided temporomandibular joint injection: Validation of an in-plane longitudinal approach	Champs B., 2019 [43]	PMID: 30412740	Wrong population (cadaveric study; no clinical TMJ patient population)
Outcomes of modified arthrocentesis using concentric needle and cannula technique with sequential viscosupplementation and orthobiologics in both TMJ compartments	Januzzi E., 2025 [39]	PMID: 40721423	Wrong intervention (ultrasound not used for real-time procedural guidance)
Use and accuracy of US guidance for image-guided injections of the temporomandibular joints in children with arthritis	Parra DA, 2010 [44]	PMID: 20204611	Wrong intervention (not exclusively US-guided; CT confirmation used)
Is Intra-Articular Steroid Injection to the Temporomandibular Joint for Juvenile Idiopathic Arthritis More Effective and Efficient When Performed with Image Guidance?	Resnick CM, 2017 [45]	PMID: 28718441	Wrong intervention (not exclusively US-guided, fluoroscopy-assisted procedure)
Does Guidance Technique Influence Success? A Double-Blind Randomized Controlled Trial Comparing the Precision and Efficacy of Image-Guided and Palpation-Guided PRP Therapy for TMJ Disorders	Pazhanivel K, 2026 [46]	PMID: 41890379	Wrong intervention (not exclusively US-guided; fluoroscopic confirmation used)
Ultrasound-guided arthrocentesis for condylar head fracture: a technical report	Hemmi T., 2024 [47]	PMID: 38568392	Wrong population (traumatic condition)
Viscosupplementation in the upper and lower compartments of the temporomandibular joint checked by ultrasonography in an ex vivo and in vivo study	Januzzi E., 2022 [48]	PMID: 36289252	Wrong intervention (ultrasound used for verification, not real-time guidance)
Comparative Analysis of the Therapeutic Effect of Ultrasound-Guided lavage and Traditional Lavage for the Treatment of Temporomandibular Joint Disorders	Xu F., 2025 [49]	https://doi.org/10.1016/j.ijom.2025.04.935	Wrong study type (conference abstract only, no full text/full data available)
Does Ultrasound Guidance Improve Effectiveness for Intra-Articular TMJ Steroid Injections in Juvenile Idiopathic Arthritis?	Resnick C.M., 2016 [50]	https://doi.org/10.1016/j.joms.2016.06.035	Wrong study type (conference abstract only)
Ultrasound-Guided Lower Compartment Injection of the Temporomandibular Joint	Lv S., 2025 [51]	https://doi.org/10.1016/j.identj.2025.104334	Wrong study type (conference abstract only)
Ultrasound-guided TMJ arthrocentesis	Stea S., 2016 [52]	https://doi.org/10.1016/j.bjoms.2016.11.233	Wrong study type (conference abstract only, no full text/full data available)
Ultrasound-guided lateral pterygoid muscle botulinum toxin: an injection for recurrent temporomandibular joint dislocation in a brain injury patient	Guo, HJ., 2022 [53]	PMID: 35486190	Wrong population (traumatic brain injury patient with neurogenic recurrent TMJ dislocation, not a typical TMJ disorder population eligible for this review)
Effectiveness of ultrasound guidance in arthrocentesis with PRP for TMJ internal derangements: A randomized controlled trial	Dolunay U., 2026 [36]	PMID: 41931266	Full text not accessible (eligibility could not be confirmed)
The Effectiveness of Ultrasound-Guided Temporomandibular Joint Injection in Reduction of Complications Rate Associated With 50% Hypertonic Prolotherapy: Comparative Study	Hassan TAL, 2026 [54]	PMID: 41556986	Full text not accessible (eligibility could not be confirmed)

Abbreviations: CT, computed tomography; PRP, platelet-rich plasma; TMJ, temporomandibular joint; US, ultrasound.

**Table A2:** JBI checklist for randomized controlled trials.

Question	Gencer 2014 [20]	Kheder 2024 [21]	Pandey 2025 [26]	Bahaa 2025 [32]	Antony 2019 [33]	Sivri 2016 [29]
True randomization?	Yes	Yes	Yes	Yes	Yes	Yes
Concealment of allocation?	Unclear	Unclear	Unclear	Yes	Unclear	Unclear
Similarity of groups at baseline?	Yes	Yes	Yes	Yes	Yes	Yes
Blinding of participants?	Yes	Unclear	Unclear	Yes	Unclear	Unclear
Blinding of those delivering treatment?	Yes	Unclear	Unclear	Yes	No	No
Blinding of outcome assessors?	Yes	Unclear	Unclear	Yes	Unclear	Unclear
Complete follow-up?	Yes	Yes	Yes	Yes	Yes	Yes
Intention-to-treat analysis?	*Yes*	*No*	*No*	*Yes*	*No*	*No*
Similar measurement of outcomes?	Yes	Yes	Yes	Yes	Yes	Yes
Appropriate statistical analysis?	Yes	Yes	Yes	Yes	Yes	Yes

**Table A3:** JBI checklist for quasi-experimental studies.

Question	Al-Delayme 2017 [23]	Othman 2026 [28]
Clear cause and effect?	Yes	Yes
Participants similar?	Yes	Yes
Similar treatment (except exposure)?	Yes	Yes
Multiple pre/post measurements?	Yes	Yes
Complete follow-up?	Yes	Yes
Reliable measurement of outcomes?	Moderate	Moderate
Appropriate statistical analysis?	Yes	Yes

**Table A4:** JBI checklist for case series.

Question	Habibi 2012 [19]	Lee 2024 [24]	Bilgir 2020 [31]	Gagnani 2020 [25]
Clear inclusion criteria?	Moderate	No	Moderate	No
Condition measured standardly?	Yes	Moderate	Yes	Moderate
Valid identification of condition?	Yes	Moderate	Yes	Moderate
Consecutive inclusion?	Unclear	Unclear	Unclear	Unclear
Complete inclusion?	Unclear	Unclear	Unclear	Unclear
Clear demographics?	Yes	Moderate	Yes	Moderate
Clear clinical information?	Moderate	No	Moderate	No
Clear reporting of outcomes?	Yes	Moderate	Yes	No
Clear site/clinic info?	Moderate	Moderate	Moderate	No
Appropriate statistical analysis?	No	No	No	No

**Table A5:** JBI checklist for text and opinion/technical reports.

Question	Tesch 2024 [27]	Levorova 2015 [30]	Dayisoylu 2013 [35]
Clear source of opinion?	Yes	Yes	Yes
Standing/position of author clear?	Yes	Yes	Yes
Interests declared?	Unclear	Unclear	Unclear
Logical argument?	Yes	Yes	Yes
Relevant to current knowledge?	Yes	Yes	Yes
